# The nitric oxide synthase gene negatively regulates biofilm formation in *Staphylococcus epidermidis*

**DOI:** 10.3389/fcimb.2022.1015859

**Published:** 2022-11-03

**Authors:** Jiaxue Wang, Lulin Rao, Zhuoan Huang, Lili Ma, Tian Yang, Zhongqi Yu, Aihua Sun, Yumei Ge

**Affiliations:** ^1^ School of Laboratory Medicine and Bioengineering, Hangzhou Medical College, Hangzhou, Zhejiang, China; ^2^ Department of Clinical Laboratory, Laboratory Medicine Center, Zhejiang Provincial People’s Hospital (Affiliated People’s Hospital, Hangzhou Medical College), Hangzhou, Zhejiang, China; ^3^ Key Laboratory of Biomarkers and In Vitro Diagnosis Translation of Zhejiang province, Hangzhou, Zhejiang, China; ^4^ Institute of Clinical Microbiology, Hangzhou Medical College, Hangzhou, Zhejiang, China; ^5^ Department of basic medicine, Hangzhou Medical College, Hangzhou, Zhejiang, China

**Keywords:** nitric oxide synthase gene, *S. epidermidis*, nitric oxide, biofilm, chronic infection

## Abstract

*Staphylococcus epidermidis (S. epidermidis)* is a clinically important conditioned pathogen that can cause a troublesome chronic implant-related infection once a biofilm is formed. The nitric oxide synthase (*NOS*) gene, which is responsible for endogenous nitric oxide synthesis, has already been found in the genome of *S. epidermidis*; however, the specific mechanisms associated with the effects of *NOS* on *S. epidermidis* pathogenicity are still unknown. The purpose of the current study was to investigate whether the *NOS* gene has an impact on biofilm formation in *S. epidermidis*. Bioinformatics analysis of the *NOS* gene was performed, and homologous recombination was subsequently employed to delete this gene. The effects of the *NOS* gene on biofilm formation of *S. epidermidis* and its underlying mechanisms were analyzed by bacterial growth assays, biofilm semiquantitative determination, Triton X-100-induced autolysis assays, and bacterial biofilm dispersal assays. Additionally, the transcription levels of *fbe*, *aap*, *icaA*, *icaR* and *sigB*, which are related to biofilm formation, were further investigated by qRT-PCR following *NOS* deletion. Phylogenetic analysis revealed that the *NOS* gene was conserved between bacterial species originating from different genera. The *NOS* deletion strain of *S. epidermidis* 1457 and its counterpart were successfully constructed. Disruption of the *NOS* gene resulted in significantly enhanced biofilm formation, slightly retarded bacterial growth, a markedly decreased autolysis rate, and drastically weakened bacterial biofilm dispersal. Our data showed that the *fbe*, *aap* and *icaA* genes were significantly upregulated, while the *icaR* and *sigB* genes were significantly downregulated, compared with the wild strain. Therefore, these data strongly suggested that the *NOS* gene can negatively regulate biofilm formation in *S. epidermidis* by affecting biofilm aggregation and dispersal.

## Introduction

*Staphylococcus epidermidis* (*S. epidermidis*), which belongs to a family of coagulase-negative *staphylococci* (CoNS), has been the leading cause of implant-associated infections in recent years (Rogers et al., 2009; Sievert et al., 2013). The main factor related to the invasiveness of this bacterium is its ability to form biofilms on implanted devices. A biofilm is a microbial community that adheres to the surface of living or inanimate objects and is surrounded by extracellular macromolecules (Otto, 2018). Since bacteria embedded in biofilms are able to resist clearance by host immune systems and antibiotics, implant-associated infections are difficult to cure and thus cause delayed healing, which places a heavy economic and psychological burden on patients (Gomes et al., 2014; Otto, 2014; Høiby et al., 2010). Whereas bacterial biofilms may be an important obstacle to bacterial infection treatment due to their higher tolerance to antibiotic treatment, the investigation of biofilm formation from a new perspective is urgently needed. The process of biofilm formation includes the initial adhesion, growth and maturation stage (also known as aggregation) as well as dispersal stages (Le et al., 2018), which can be regulated by different types of molecules, such as extracellular polymers (PIAs), autolysin and cell wall-anchoring proteins (Vuong et al., 2004; Otto, 2008; Conlon et al., 2014). Although the mechanisms of biofilm regulation have been studied extensively in multiple other bacteria, such as *Staphylococcus aureus*, *Pseudomonas aeruginosa* and *Escherichia coli*, the regulation of biofilm formation in *S. epidermidis* has appeared to be more important, as biofilms are one of its most significant virulence factors.

Nitric oxide (NO) has been established as a well-known bioactive gaseous molecule that has been extensively studied in animals and plants in recent years. It has been described that NO plays an important role in human host defense against pathogen infection and immune modulation (Lee et al., 2017). Previous studies have demonstrated that NO can not only exert some essential biological functions in microorganisms but also regulate bacterial biofilm formation (Kim et al., 2017) and drug resistance (Gusarov et al., 2009). Additionally, endogenous bacterial NO can also contribute to protecting bacteria against oxidative stress, such as antibiotic-induced oxidative stress (Holden et al., 2015), thus demonstrating that it may participate in bacterial resistance. The *NOS* gene, which is responsible for the synthesis of NO (Napoli et al., 2013), is universally distributed throughout animal, plant and microbial genomes (Holden et al., 2015; Holden et al., 2016). Multiple studies have demonstrated that bacterial nitric oxide synthase (bNOS) may be regarded as a potential drug target, considering that it provides bacteria with a protective mechanism similar to NO functions (Shatalin et al., 2008; Gusarov et al., 2009; van Sorge et al., 2013). In addition, it has been observed that bNOS is conducive to enhancing bacterial colonization and virulence (Sapp et al., 2014; Kinkel et al., 2016). Interestingly, in our previously published study, a positive correlation was observed between the *ArlRS* two-component regulatory system, which can significantly promote biofilm formation of *S. epidermidis*, and *NOS* expression, suggesting that *NOS* is probably involved in biofilm formation in *S. epidermidis* (Wu et al., 2012). In view of some critical roles of biofilms in *S. epidermidis*, the impact of the *NOS* gene on biofilm formation warrants investigation. If any important functions of *NOS*, such as its significant impact on a certain stage of biofilm formation, are identified in the current study, it will be beneficial to deeply understand biofilm-related infections of *S. epidermidis*, thus providing a theoretical basis for the control of this type of infection.

In this study, the *NOS* gene of *S. epidermidis* was deleted and complemented. The effect of *NOS* deletion on biofilm formation was observed, and the regulation of biofilm formation was further explored. Here, we first describe the key roles of the *NOS* gene in regulating biofilm formation of *S. epidermidis*, which is mainly reflected in its impact upon the stage of biofilm aggregation and dispersal.

## Materials and methods

### Strains, plasmids and bacterial growth conditions

The strains and plasmids used in this study were kindly provided by Dr. Yang Wu from Shanghai Medical College, Fudan University, and were as follows: *S. epidermidis* strain 1457 (Genome Accession Number: NZ_CP020463.1), *Escherichia coli* (*E. coli*) strain DC10B and plasmid pKOR1 for the purpose of gene replacement, as well as shuttle vector pRB473 for the construction of complement strain. *E. coli* was routinely grown in Luria-Bertani (LB) medium (Bio-Rad, USA). Basic medium (BM medium) and tryptic soy broth (TSB) medium (OXOID, England) were used for cultivation of *S. epidermidis*. When necessary, antibiotics were added to the medium at a final concentration of 50 μg/ml ampicillin for *E. coli* and 50 ng/ml anhydrotetracycline and 10 or 5 μg/ml chloramphenicol for *S. epidermidis* (Sigma−Aldrich, USA). Unless otherwise specified, bacterial cultures were incubated at 37°C with shaking at 220 r.p.m.

### Construction of phylogenetic tree and evaluation

The nucleotide sequences of the *NOS* gene of *S. epidermidis 1457* were verified and utilized to perform phylogenetic analysis by Molecular Evolutionary Genetics Analysis (MEGA) software version 11. *Staphylococcus* sp., *E. coli* and *Bacillus subtilis* were selected for evolutionary tree analysis of the *NOS* gene.

### Construction of the *NOS* deletion strain

For generation of the *NOS* mutant, targeted deletion of the *NOS* gene was performed by a Gateway-compatible allelic exchange system as previously described with minor modification (Bae and Schneewind, 2006). Briefly, two regions with sizes of 1032 bp and 1012 bp located upstream and downstream of the *NOS* gene, respectively, were amplified using the primers listed in **Table 1**. The targeting fragments were ligated by fusion PCR, cloned into the temperature-sensitive shuttle plasmid pKOR1 using BP Clonase™ II Enzyme Mix (Invitrogen) according to the manufacturer’s instructions, and then transformed into *E. coli* strain DC10B lacking cytosine methylation (DH10B Δ*dcm*), yielding the recombinant plasmid pKOR1-Δ*NOS*. The isolated plasmid was then electroporated directly into *S. epidermidis* strain 1457 by a Gene Pulser Xcell Electroporater (Bio-Rad, USA) due to its capacity to bypass the type IV restriction barrier. To promote integration of pKOR1-Δ*NOS* into the chromosome of *S. epidermidis*, one transformant was cultivated at 30°C in TSB medium containing 10 μg/mL chloramphenicol (TSB_Cm10_) and then subcultured at the nonpermissive temperature of 43°C for two rounds. The subculture aliquots were plated onto TSB_Cm10_ agar and incubated at 43°C for 18-24 h. To increase the efficiency of homologous recombination, integrant colonies were chosen by colony PCR to determine whether single crossover had occurred upstream (through the primers *NOS*-IF and *NOS*-DR) or downstream (through the primers *NOS*-UF and *NOS*-IR) of the target gene. For counterselection, 18-24-h cultures of the confirmed integrant were subsequently aerated at 37°C without antibiotics and then diluted and homogenized onto TSB agar containing 50 ng/mL anhydrotetracycline (Sigma−Aldrich, USA). Large colonies were picked up and inoculated onto antibiotic-free TSB agar and TSB_Cm10_ agar and then incubated at 37°C for 18-24 h. To obtain the desired knockout mutant (SE1457*-ΔNOS)*, PCR screening of chloramphenicol-sensitive colonies was performed using the primers *NOS*-IF and *NOS*-IR. All primers used in this study were designed and synthesized by Clone Manager 8.0 software (Sci Ed Software LLC., USA) and Sangon Biotech (Shanghai, China) Co., Ltd., respectively.

**Table 1:** Primer sequences used in this study.

Primer names	Primer sequences (5’-3’)			Amplified fragments(bp)
**Primers used as *NOS* deletion and reversion**				
*NOS*-up-F	5’-GGGGACAAGTTTGTACAAAAAAGCAGGCTCAGCCTCATCTAACATTCTA-3’(attB1)			1032
*NOS*-up-R	5’-ATTATGCCACCTCGTTTAAATTAAAACACCTCAATCGTCA-3’			
*NOS*-down-F	5’-TGACGATTGAGGTGTTTTAATTTAAACGAGGTGGCATAAT-3’			1012
*NOS*-down-R	5’-GGGGACCACTTTGTACAAGAAAGCTGGGTCTTATATAGGCTGAAGCTGT-3’(attB2)		
Up-screen-F	5’-CTTGTAATAGTCTGCTCATC-3’			3541
Down-screen-R	5’-AGATGCTTCACAATCAATAC-3’		
*NOS*-CF	5’-CCGGAATTCAGGTACTTGCATGTTTGATTTACA-3’		*EcoRI*	1223
*NOS*-CR	5’-CGCGGATCCTGCATTATGCCACCTCGTTT-3’		*BamH I*
**Primers uses as qRT-PCR**				
*gyrB* (internal gene)	*gyrB*-qF	5’-TAGTATTGACGAGGCATTAGCA-3’		263
	*gyrB*-qR	5’-TATCCGCCACCTCCGA-3’	
*icaA*	*icaA*-qF	5’-TGCACTCAATGAGGGAATCA-3’		205
	*icaA*-qR	5’-TAACTGCGCCTAATTTTGGATT-3’	
*aap*	*aap*-qF	5’-GCACCAGCTGTTGTTGTACC-3’		184
	*aap*-qR	5’-GCATGCCTGCTGATAGTTCA-3’	
*fbe*	*fbe*-qF	5’-TTGAAGCCAGGCATAACG-3’		177
	*fbe*-qR	5’-TAAACACCTTGAGGGAGG-3’	
*icaR*	*icaR*-qF	5’-GGAGCACTAGATAATTGAACAT-3’		268
	*icaR*-qR	5’-CATTGACGGACTTTACCAG-3’	
*sigB*	*sigB*-qF	5’-TCACCTGAACAAATTAACCAATG-3’		259
	*sigB*-qR	5’-CACCTATTAGACCAACCATACC-3’	

### Generation of SE *1457-ΔNOS* complementation strain

The pRB473 plasmid was used to construct the complementation strain. The full-length *NOS* gene, including its promoter region and ribosome binding site, was amplified using *SE1457* genomic DNA as the template and ligated into the vector pRB473 to construct the complementation plasmid *pRB473-NOS*. The complementation plasmid was identified through restriction enzyme digestion (Takara, Japan), PCR and sequencing and then transformed into *SE1457-ΔNOS* to obtain the complementation strain, named *SE1457-ΔNOS:cNOS*.

### Bacterial biofilm formation assay

Twenty-four-hour cultures were diluted with TSB medium (containing 0.5% glucose) at a ratio of 1:200, inoculated into a 96-well microtiter plate (Costar, USA), and incubated statically at 37°C for 6, 12, 24 and 48 h. The bacterial culture was discarded, and nonadherent cells were washed three times with PBS. The biofilm was fixed with 99% methanol and stained with 2% crystal violet for 15 min. The culture plates were rinsed and dried at room temperature. Finally, OD_570_ values were determined by a SpectraMax 190 Microplate Reader (MD, USA). Experiments were repeated at least three times (Zhu et al., 2017).

### Determination of bacterial growth curves and observation of colony morphology

To obtain the bacterial growth curves, 24 h cultures were diluted to an OD_600_ of 0.01 in fresh TSB medium and incubated at 37°C with shaking at 220 r.p.m. The turbidity was measured continuously for 12 h at an interval of one hour by a SmartSpec 3000 Spectrophotometer (Bio-Rad, USA) (Zhu et al., 2017). According to the conventional approach, 24-h cultures were diluted 1:15000 and subsequently coated onto TSB plates to observe the colony morphology. After incubation at 37°C for 18 h, the colony sizes of the SE1457, SE1457-*△NOS* and SE1457-*△NOS:cNOS* strains were compared.

### Triton X-100 induced bacterial autolysis

Triton X-100-induced autolysis experiments were carried out as previously described (Zhu et al., 2017). Briefly, the bacteria were grown in TSB medium containing 1 M NaCl until the mid-logarithmic growth phase (OD_600_ = 0.6-0.8) and then centrifuged and washed with precooled deionized water. The harvested cell pellets were resuspended in Triton X-100 autolysis buffer (Sigma−Aldrich, USA; 50 mM glycine, pH 8.0, containing 0.01% Triton X-100) and incubated with shaking at 30°C. The autolysis was monitored by measuring the decrease in OD_600_ of bacterial suspensions every 30 min for 6 h by a SmartSpec 3000 Spectrophotometer (Bio-Rad, USA). Autolysis curves were generated, and statistical analysis was performed using SPSS 13.0 software. All experiments were conducted using at least three biological replicates.

### Biofilm dispersal assay of* S. epidermidis*

To determine the biofilm dispersal capacity, appropriately modified methods have been carried out (Boles and Horswill, 2008; Schreiber et al., 2011; Zemke et al., 2020). In brief, the bacterial concentration was adjusted to OD_600_ = 0.2-0.3 with TSB medium, and a sufficient dilution of the bacterial solution was then performed with TSB containing 0.5% glucose (TSBG). The diluted bacterial solution was added to a 96-well plate and incubated at 37°C for 48 h to form a stable colony biofilm. Then, 20 colony biofilm wells were randomly selected. Subsequently, 100 μL of TSB medium was added to each colony separately and incubated for approximately one hour at room temperature. Immediately, 80 μL of medium was gently aspirated from each colony without any mixing, subsequently coated on a TSA plate and incubated at 37°C for 18-24 h after being diluted appropriately, and the colony count per plate was calculated. Finally, the biofilm dispersal capacity was determined according to the bacterial count from the original incubation solution, and the calculation formula was as follows: biofilm dispersal capacity = colony number (CFU) per plate × dilution multiple. The experiment was repeated at least three times.

### Bacterial RNA isolation and qRT−PCR

Gene expression of *NOS* will arrive at the highest level after incubation for approximately 10 h according to our previous experiments. Therefore, 10 h after incubation was regarded as the appropriate time point for determining *NOS* expression. Sodium nitroprusside (SNP) alone and the SNP + 2-phenyl-4,4,5,5-tetramethyl-imidazoline-1-oxyl-3-oxide (PTIO) combination were used to determine the expression level of *NOS*. Bacterial cultures were inoculated for 10 h in TSB (0.5% glucose). Subsequently, the bacterial culture was centrifuged (12000 r.p.m, 4°C for 15 min), and the cell pellets were resuspended in RNA-free PBS solution. However, with regard to RNA extraction for the expression analysis of biofilm-related genes, the selected time points were different, namely, 4, 6, 8, 10 and 12 h after culture. RNA extraction and reverse transcription were performed according to the instruction manual of the RNA Extraction Kit (Takara, Japan). The fluorescent quantitative PCR conditions were as follows: SYBR Green 5 μl, sense primer 0.5 μl, antisense primer 0.5 μl, cDNA template 1.2 μl, and dd H_2_O 2.8 μl. Then, 30 cycles of 95°C for 30 s, 95°C for 6 s, and 59°C for 6 s were performed. The relative expression of each gene was calculated using the Livak method (2^-△△Ct^) as described previously (Lewis and Rice, 2016). The primers used for quantitative PCR are listed in **Table 1**. For each gene transcript, qPCR was performed with three independent samples and three technical replicates for each sample.

To accurately prepare the appropriate concentrations of SNP and PTIO, which were used as NO donors and scavengers, respectively, one gram of SNP (Sigma−Aldrich, USA) and 5 ml of deionized water were added to obtain a 200 mg/ml stock solution after filtration (0.45 μm). Subsequently, working solutions of 0.25, 0.5 and 1.0 mol/L were prepared on this basis. According to the same method as described above, 1 g of PTIO was weighed (Sigma−Aldrich, USA), 50 ml of deionized water was added to obtain a 20 mg/ml stock solution, and a 1.0 mol/L working solution was finally prepared. All prepared reagents were stored in a -20°C refrigerator for later use.

### Statistical analysis

Data are expressed as the mean ± standard deviation of three independent experiments. Statistical analysis was performed using SPSS 13.0 software, and statistical significance was determined by a paired Student’s t test (*p value* < 0.01).

## Results

### Nitric oxide donor (SNP) inhibits *NOS* gene expression and biofilm formation in *S. epidermidis*

**Figure 1:**
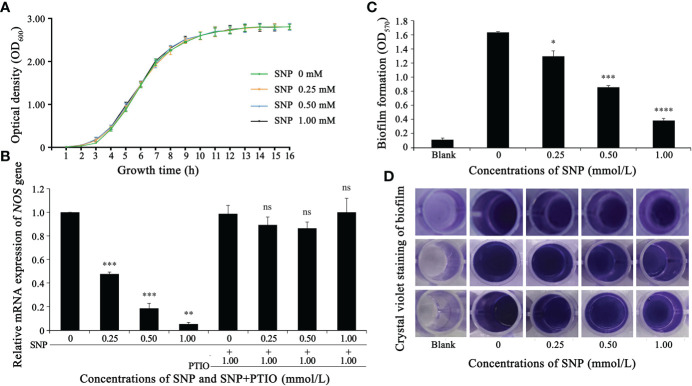
Nitric oxide donor (SNP) inhibits *NOS* gene expression and biofilm formation in *S. epidermidis*. **(A)** SNPs of 0, 0.25, 0.5 and 1 mM exhibited no significant difference in the growth of *S. epidermidis* (p > 0.1). **(B)** The effect of SNP on the *NOS* gene was analyzed through fluorescent quantitative PCR (2^-△△Ct^ method). **(C, D)** The effect of SNP on the biofilm formation of *S. epidermidis* was analyzed by crystal violet staining. Experimental data are presented as the mean ± SD obtained from three independent experiments. *p < 0.05, **p < 0.01, ***p < 0.001, ****p < 0.0001, ns, not significant.

To verify the hypothesis that SNP does not affect the growth of *S. epidermidis*, we conducted a bacterial growth test and measured the growth curve of *S. epidermidis* under different concentrations of SNP. As shown in **Figure 1A**, SNP at 0 mM, 0.25 mM, 0.5 mM and 1 mM had no significant effect on the growth of *S. epidermidis*.

Effects on biofilm biomass and planktonic biomass were determined by adding a range of concentrations of NO donor SNP into *S. epidermidis* cultures. Biofilm biomass and planktonic biomass displayed an apparent decrease and increase in millimolar concentration ranges, respectively. We further found that the greatest effect could be observed repeatedly when 0.5 mM SNP was used, which showed that the ratio of biofilm to planktonic cells was decreased by nearly 10-fold (data unpublished). Thus, the concentration of 0.5 mM SNP was selected as a basis for further experiments. To explore the relationship between SNP and *NOS* expression levels, SNP at a final concentration between 0.25-1.0 mmoL/L was added to *S. epidermidis* cultures that had been cultivated for 10 h, and total bacterial RNA was then extracted for subsequent qRT-PCR analysis. The transcript level of the *NOS* gene gradually decreased with increasing SNP concentration, while PTIO, which can act as a nitric oxide scavenger, significantly restored its level (**Figure 1B**). Additionally, the effect of SNP on biofilm formation of *S. epidermidis* was investigated by 96-well plate crystal violet staining. We found that biofilm-forming ability gradually attenuated with increasing SNP concentration (**Figures 1C**, **D**). These results, combined with previous observations, strongly suggest that the *NOS* gene may regulate the biofilm formation of *S. epidermidis*.

### Phylogenetic tree and evaluation of the NOS gene in *S. epidermidis 1457*

**Figure 2:**
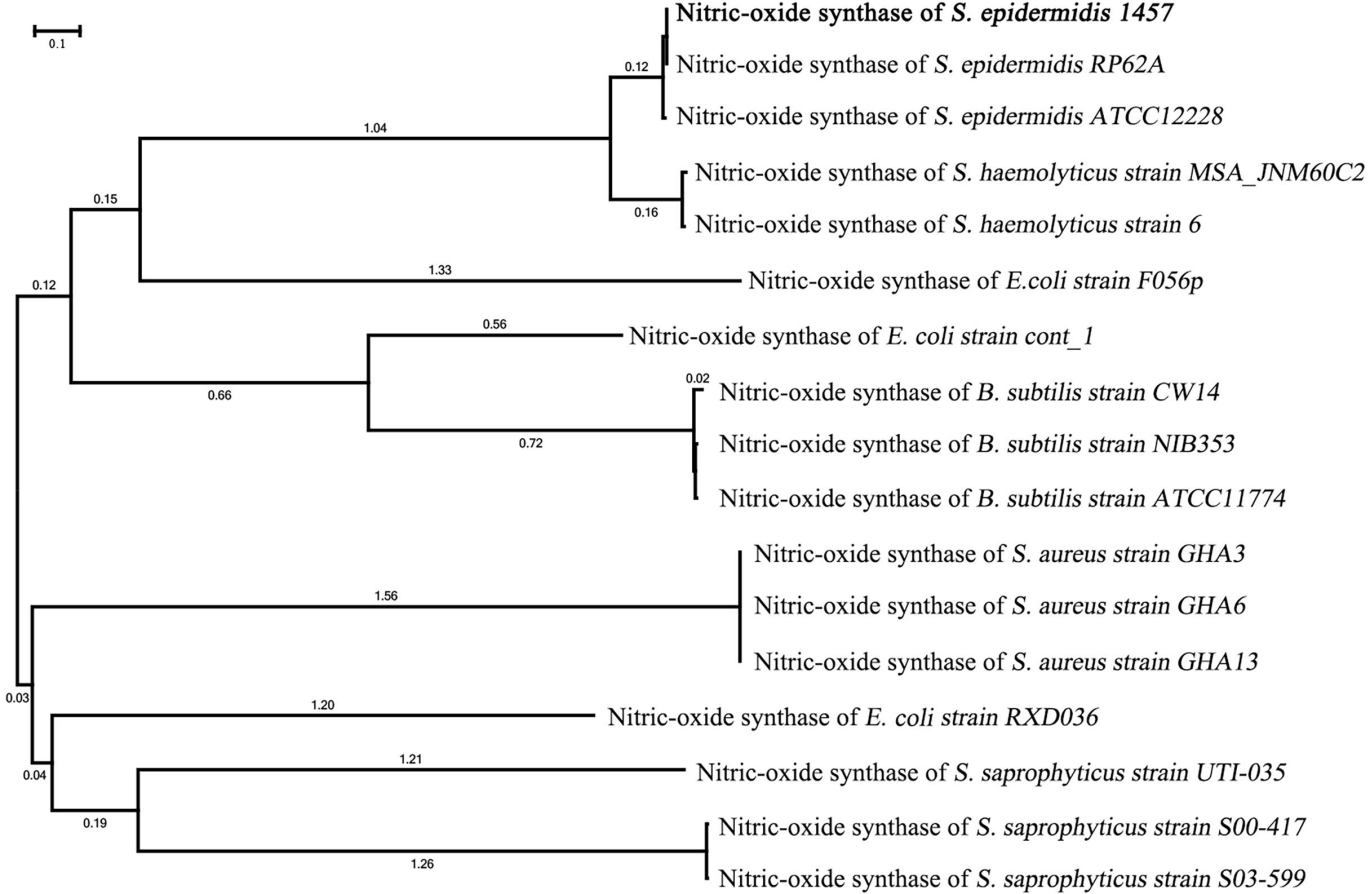
Phylogenetic tree of the *NOS* gene in *S. epidermidis 1457.* The phylogenetic tree of *NOS* of *S. epidermidis* 1457 showed that it had the closest homology with NOS of *S. epidermidis* RP62A and *S. epidermidis* ATCC 12228.

The phylogenetic tree of *NOS* of *S. epidermidis* 1457 showed that it had the closest homology with *NOS* of *S. epidermidis* RP62A (100%) and *S. epidermidis* ATCC 12228 (98.9%). The nucleic acid sequence of *NOS* in *S. epidermidis* 1457 revealed 95.2% similarity with *S. hemolyticus strain MSA_JNM60C*, 94.8% similarity with *S. hemolyticus strain 6*, 88.3% similarity with *E. coli strain F056p*, 84.4% similarity with *E. coli strain cont_1*, 81.5% similarity with *B. subtilis strain CW14*, 80.6% similarity with *B. subtilis strain NIB353*, 80.9% similarity with *B. subtilis strain ATCC11774*, 77.2% similarity with *S. aureus strain GHA3*, 77.9% similarity with *S. aureus strain GHA6*, 76.4% similarity with *S. aureus GHA13*, 75.1% similarity with *E. coli strain RXD036*, 74.6% similarity with *S. saprophyticus strain UTI-035*, 72.1% similarity with *S. saprophyticus strain S00-417*, and 71.9% similarity with *S. saprophyticus strain S03-599* (**Figure 2**). The results indicate that the *NOS* gene is conserved between bacterial species originating from different genera.

### *NOS* deletion impacts *S. epidermidis* aerobic growth

**Figure 3:**
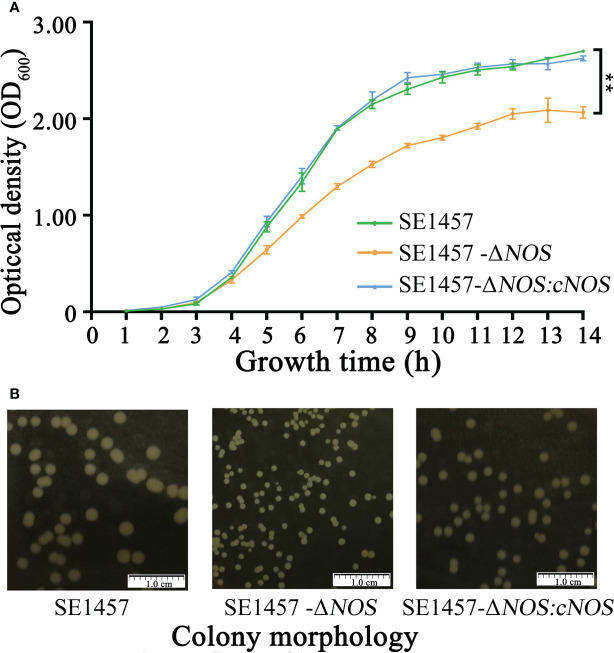
Determination of the bacterial growth curves and observation of bacterial colony morphology. **(A)** The bacterial growth ability was significantly attenuated following *NOS* deletion. **(B)** *NOS* deletion apparently affected the size of bacterial colonies. *SE1457*: the wild-type strain; *SE1457-ΔNO*S: the deletion strain; *SE1457-ΔNOS: cNO*S: the complementation strain. These curves represent three independent experiments. **p < 0.01.

To verify the hypothesis that the *NOS* gene has an effect on the growth of *S. epidermidis*, bacterial growth tests were carried out, including the determination of growth curves and observation of colony morphology. The SE1457-*ΔNOS* strain displayed slower growth than the wild strain, and its growth rate began to show a steady decline at the logarithmic phase of approximately 4 h. Moreover, the OD_600_ value of bacterial growth after reaching the plateau phase was also maintained at a level lower than that of the wild strain, while the complementation and wild strains had comparable growth patterns, suggesting that *NOS* gene complementation can significantly restore the growth ability of bacteria (**Figure 3A**). The colony morphology was also assessed on aerobic tryptic soy agar (TSA) plates for SE1457-*ΔNOS* and its isogenic mutant. At 24 h of growth, the colony morphology of the SE1457-*△NOS* mutant was significantly smaller than that of the SE1457 wild-type strain, and the phenotype could be partially complemented in the SE1457-*ΔNOS* complementation strain (**Figure 3B**).

### Effect of *NOS* deletion on bacterial biofilm formation

**Figure 4:**
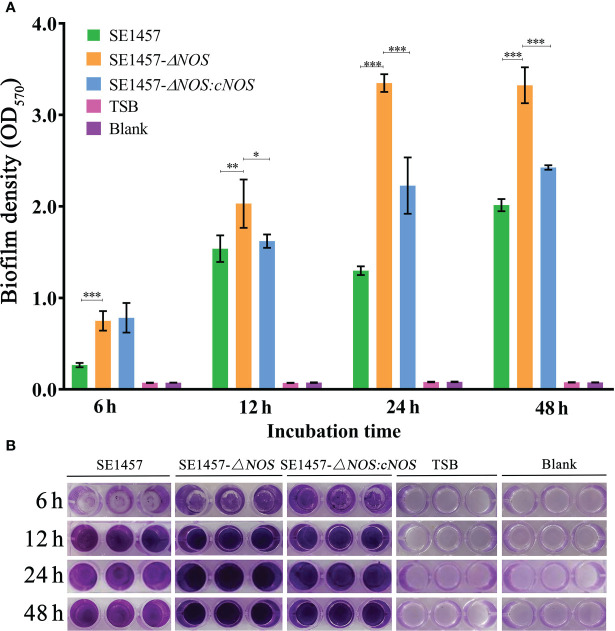
Determination of bacterial biofilm formation. Experimental data are presented as the mean ± SD obtained from three independent experiments. *SE1457*, *SE1457-ΔNOS* and *SE1457-ΔNOS:cNOS* are the wild strain, deletion strain and complement strain, respectively. *p < 0.05, **p < 0.01, ***p < 0.001. **(A)** Semi-quantitative determination of the biofilm formation of S. epidermidis at different culture time points. **(B)** Qualitative determination of the biofilm formation of S. epidermidis by crystal violet staining.

To investigate whether *NOS* deletion affects the biofilm formation of *S. epidermidis*, bacterial biofilm formation ability was semiquantitatively determined by using crystal violet staining. Cultures cultivated for 6, 12, 24 and 48 h were selected for biofilm determination. The results showed that the values of biofilm formation by *SE1457* (OD_570_) were 0.27 ± 0.02, 1.54 ± 0.15, 1.23 ± 0.048, and 2.01 ± 0.07, respectively. However, the values of biofilm formation by the *SE1457- ΔNOS* strain were 0.75 ± 0.11, 2.03 ± 0.26, 3.34 ± 0.10, and 3.32 ± 0.20, respectively. The values formed by the *SE1457-ΔNOS:cNOS* strain were 0.78 ± 0.17, 1.62 ± 0.07, 2.22 ± 0.30, and 2.43 ± 0.02, respectively. Bacterial biofilm formation ability was significantly enhanced after *NOS* deletion compared with the wild-type strain (*p* < 0.001, *p* < 0.01, *p* < 0.001, *p* < 0.001, respectively), and the difference was maximized at 24 h (**Figure 4A**). The biofilm formation of the complemented strain was weaker than that of the deletion strain at the remaining time points except for 6 h (*p* < 0.05, *p* < 0.05, *p* < 0.001, and *p* < 0.001, respectively), indicating that the bacterial biofilm formation ability was reduced to some extent after gene reversion (**Figure 4B**).

### Impact of *NOS* deletion on *S. epidermidis* autolysis

**Figure 5:**
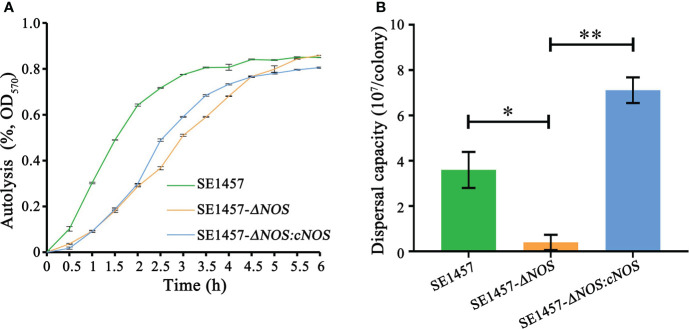
Determination of the bacterial autolysis induced by 0.01% Triton X-100 and bacterial biofilm dispersal capacity. **(A)** Experimental data are presented as the mean ± SD obtained from three independent experiments. *SE1457*, *SE1457-ΔNOS* and *SE1457-ΔNOS:cNOS* are the wild strain, deletion strain and complement strain, respectively. **(B)** Experimental data are presented as the mean ± SD obtained from three independent experiments. *SE1457*, *SE1457-ΔNOS* and *SE1457-ΔNOS:cNOS* are the wild strain, deletion strain and complement strain, respectively. *p < 0.05, **p < 0.01.

The autolysis of bacteria can be induced by Triton X-100. To detect the influence of *NOS* deletion on bacterial autolysis activity, we evaluated the autolysis ability of *S. epidermidis* by the Triton X-100 induction method in this study. The results showed that the autolysis rate of the deletion strain was slower than that of the wild-type strain under the induction of 0.01% Triton X-100; however, the final autolysis rate of both strains was consistent (80.25%) after 6 h of induction. Compared with the deletion strain, the autolysis rate of the complement strain was accelerated at approximately 2-4.5 h, indicating that the bacterial autolysis ability was restored to some extent after gene complementation. More surprisingly, the autolysis rate of the complement strain was slightly decreased compared with that of the wild-type and deletion strains after approximately 6 h of induction (**Figure 5A**).

### Effect of *NOS* deletion on the *S. epidermidis* biofilm dispersal capacity

Once a suitable opportunity exists, bacteria can break their biofilm bonds and migrate to new environments to form new biofilms, thereby resulting in disseminated infection (Guilhen et al., 2017). Therefore, it is very significant to understand the impact of *NOS* deletion on the *S. epidermidis* biofilm dispersal capacity, so we determined the bacterial biofilm dispersal capacity in the wild-type, mutant and complemented strains. The results showed that the bacterial biofilm dispersal ability of the deletion strain was reduced compared with that of the wild-type strain (*p* < 0.05), with average numbers of disseminated bacteria of 0.4 × 10^7^ cfu/colony and 3.6 × 10^7^ cfu/colony, respectively. However, the bacterial biofilm dispersal ability of the complemented strain was significantly enhanced compared with that of the deletion strain (*p* < 0.01), with average numbers of disseminated bacteria of 7.1 × 10^7^ cfu/colony and 0.4 × 10^7^ cfu/colony, respectively (**Figure 5B**).

### Effects of *NOS* deletion on the expression of genes related to biofilm formation

**Figure 6:**
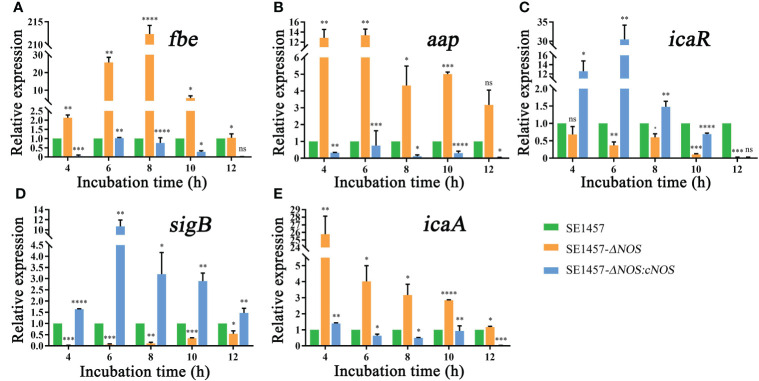
**(A)** Effects of NOS deletion on the transcriptional levels of *fbe*. **(B)** Effects of NOS deletion on the transcriptional levels of *aap*. **(C)** Effects of NOS deletion on the transcriptional levels of *icaR*. **(D)** Effects of NOS deletion on the transcriptional levels of *SigB*. **(E)** Effects of NOS deletion on the transcriptional levels of *icaA*. *p < 0.05, **p < 0.01, ***p < 0.001, ****p < 0.0001, ns, not significant.

The process of biofilm formation involves a variety of factors related to *S. epidermidis*. The fibrinogen-binding protein (*fbe*), accumulation-associated protein (*aap*), *icaR*, sigma Factor B (*sigB*) and *icaA* genes are closely related to biofilm formation of *S. epidermidis* (Conlon et al., 2002; Hennig et al., 2007; Herman et al., 2014; Xu et al., 2017). Therefore, the genes mentioned above were selected to analyze RNA transcriptional levels to observe the impact of *NOS* deletion on biofilm formation-related gene expression in *S. epidermidis* at the time points shown in **Figure 6**. As expected, the expression of the *fbe* gene from the deletion strain showed an obvious initial increase but subsequently displayed a slow decrease with extended incubation time; however, its expression in the deletion strain was obviously upregulated compared with that of the wild strain at all time points, with the maximum transcriptional level upregulated by 212.32-fold. Unsurprisingly, the transcriptional level of the complemented strain was significantly attenuated compared with that of the wild-type strain at all time points (**Figure 6A**). It was also found that the gene transcription levels of *aap* and *icaA* were generally attenuated over time and were significantly higher than those of the wild strain, with maximum upregulation by 13.37- and 25.77-fold, respectively, while the transcriptional levels of the complement strain were all lower than or similar to those of the wild strain (**Figures 6B**, **E**). However, the transcriptional levels of the *icaR* and *sigB* genes were attenuated in the deletion strain, both weakened by a maximum of 50-fold, and the transcriptional levels of the corresponding genes were markedly enhanced after gene reversion by 30.48- and 10.71-fold, respectively (**Figures 6C, D**).

## Discussion

*S. epidermidis* is becoming an important infectious pathogenic bacterium due to the increasing number of artificially implanted medical materials. Unlike *S. aureus*, which has many virulence pathogenetic factors, *S. epidermidis* lacks invasive virulence factors, and biofilms are its most important pathogenic substance. Over the last decades, an increasing number of studies have focused on biofilm formation. Bacterial biofilm formation involves several steps, which can be divided into initial attachment, maturation and biofilm dispersal, and the final step is the detachment of bacterial strains from microcolonies, potentially resulting in new biofilm colony formation in a distinct location (Kamaruzzaman et al., 2018). The biofilm formation of *S. epidermidis* involves multiple factors. For example, the aggregation-associated protein (Aap) of staphylococcal surface proteins can regulate multilayer cell clusters and filamentous protein network formation, and intercellular polysaccharide adhesion (PIA) encoded by the *icaADBC* operon is a well-studied intercellular adhesin, both of which can be involved in the biofilm formation aggregation stage (Hennig et al., 2007). In addition, *icaR* encodes a profilin of the *icaADBC* operon and has a repressive effect on the production of PIA (Xu et al., 2017). However, as an important regulating factor for the *ica* operon, *sigB* can negatively regulate the *icaR* gene. It regulates the *icaADBC* operon through an *icaR*-dependent pathway and plays a pronounced effect on biofilm formation (Conlon et al., 2002). The *S. epidermidis* serine-aspartate repeat protein G (SdrG), known as fibrinogen binding protein (Fbe), contains an LPXTG motif that can covalently link the bacterial cell wall surface and is involved in the process of bacterial adhesion to fibrinogen, which also plays a critical role in the aggregation phase of biofilm formation (Herman et al., 2014). AtlE-mediated autolysis of *S. epidermidis* can promote the release of extracellular DNA (eDNA) from bacteria, which acts as an intercellular adhesion factor to improve biofilm stability and plays an important role in both adhesion and aggregation stages (Christner et al., 2012).

NO is a well-studied biomolecule, and relevant studies have shown that bacterial endogenous nitric oxide can regulate biofilm formation and drug resistance in bacteria (Gusarov et al., 2009; Holden et al., 2015). It has been revealed that bacterial endogenous NO plays an important role in bacterial infection and has an important contribution to bacterial survival (van Sorge et al., 2013). SNPs are usually used as NO donors in microbiological experiments, and a stable concentration of NO can be obtained by adding an appropriate concentration of SNP into bacterial culture; thus, SNPs can be used to mimic endogenous bacterial NO production (Bae and Schneewind, 2006; Patel et al., 2009). NOS is a key enzyme for NO synthesis in bacteria, and it is encoded by the *NOS* gene. With the help of NOS, bacteria can utilize L-arginine as a substrate and finally generate endogenous NO, which participates in interbacterial signal transmission to exert a variety of functions (Zumft, 2005) through oxidation, dehydrogenation, cracking, hydrolysis and other reactions (Ras et al., 2018). As a feedback mechanism of bacteria, we speculated that SNP may affect *NOS* gene expression in *S. epidermidis*. Interestingly, as expected, SNP with a concentration range of 0.25-1.0 mM gradually inhibited the expression of the *NOS* gene at the RNA level, as shown in **Figure 1B**. NOS can interact with bacterial proteins and, thus, regulate bacterial pathogenesis, and endogenous NO can control bacterial biofilm formation by regulating the two-component signal transduction system (TCTS) (Rao et al., 2015). As described in **Figure 1C, D**, our results showed that the biofilm formation ability gradually attenuated with increasing SNP concentration. Therefore, the combined results described in **Figure 1** show that the *NOS* gene may be involved in the regulation of biofilm formation in *S. epidermidis*.

We hypothesized that NOS may exert some considerable functions by regulating the biofilm formation of *S. epidermidis*. To investigate this hypothesis, we successfully constructed a *NOS* deletion strain (**Figure 1** of the supplementary materials) and assessed whether and how NOS deletion affects bacterial biofilm formation in *S. epidermidis*. It was demonstrated that *NOS* deletion could result from a markedly reduced ability of bacterial growth (**Figure 3**), and biofilm formation was significantly enhanced following *NOS* deletion (**Figure 4**). Bacteria can be released from biofilms to return to a planktonic state for distant spread and recolonization (Guilhen et al., 2017), which is particularly important in the development of chronic infection. It was found that *NOS* deletion can significantly reduce the ability of bacterial biofilm dispersal of *S. epidermidis* (**Figure 5B**). In summary, the current findings reinforce that NOS is one of the factors involved in biofilm formation of *S. epidermidis*, which together suggests that it can negatively regulate bacterial biofilm formation by affecting the stage of biofilm aggregation and dispersal.

In the present study, several genes related to biofilm formation were selected to analyze their transcription levels to further explore the mechanism of NOS in regulating biofilm formation. All the selected genes showed significant changes at the transcriptional level compared with the wild-type strain (**Figure 6**). Additionally, it has been demonstrated that bacterial autolysis is associated with the *atlE* gene, which encodes autolysin AtlE, and that biofilm formation ability is significantly reduced after deletion of the *atlE* gene in another study (Qin et al., 2007). In our study, the autolysis rate of bacteria decreased significantly after *NOS* deletion. There is currently no evidence indicating the correlation and significance between *NOS* and *atlE*; thus, further investigation is warranted. Moreover, bacterial autolysis can also reflect the increased ability of bacterial biofilms to disseminate into the external environment (Qin et al., 2007). Impressively, there was a positive correlation between bacterial biofilm dispersal and autolysis, as shown in this study; unfortunately, this correlation has not been well clarified; if the correlation were to be clarified, it would help us further understand the mechanisms of biofilm formation in *S. epidermidis*. We already know that the *fbe*, *aap*, and *icaA* genes all prominently contribute to bacterial biofilm formation and play an important role in its aggregation stage (Zumft, 2005; Hennig et al., 2007), while the *icaR* gene can negatively regulate biofilm formation in *S. epidermidis* (Xu et al., 2017). It has been reported in the previous literature that the *icaR* gene could negatively regulate biofilm formation in *S. epidermidis*. Previous studies have demonstrated that the deletion of *sigB* in *S. epidermidis* leads to the upregulation of *icaR* and the reduction of biofilm formation, which confirms that *sigB* negatively regulates *icaR* (Knobloch et al., 2004). However, as described in **Figure 6**, this study showed that all these genes were significantly upregulated following *NOS* deletion, excluding *icaR* and *sigB*, which were obviously downregulated. Interestingly, *NOS* deletion resulted in decreased *SigB* expression but also decreased *icaR* expression, which seems to contradict previous studies. Although there is a negative regulatory relationship between *sigB* and *icaR*, it has been reported in other bacteria that *icaR* may also have other regulated pathways, such as the regulation of *icaR* by the quorum sensing system. If the regulation of *icaR* by the quorum sensing system exceeds the negative regulation of *icaR* by *sigB*, there will be a phenomenon in which the expression of both *sigB* and *icaR* genes decreases at the same time and the formation of biofilm increases. Dan Yu et al. confirmed that knockout of the *LuxS* gene in *S. aureus* will lead to a decrease in AI2 and, thus, *icaR*, so the biofilm synthesis of *LuxS* knockout strains is higher than that of wild strains (Yu et al., 2012). Therefore, if *icaR* is regulated by *sigB* and other quorum sensing systems at the same time, the biofilm formation ability may ultimately be enhanced. Currently, studies have shown that there is also a quorum sensing system in *S. epidermidis*. The *LuxS* and *agr* systems are important genes of the quorum sensing system of *S. epidermidis*, which can regulate the formation of bacterial biofilms (Xu et al., 2006). Plate et al. reported that heme nitrogenous oxide/oxygen binding (H-NOX) protein is a sensing receptor of no molecules, and H-NOX pathways regulate bacterial biofilm formation and quorum sensing (Plate and Marletta, 2013). More experiments are needed to confirm the specific mechanism by which the quorum sensing system regulates icaR.

NOS in *S. aureus* is associated with virulence (Surdel et al., 2016) and can also increase antibiotic resistance as well as susceptibility to oxidative stress (Kinkel et al., 2016). Additionally, NOS can also participate in bacterial metabolic processing, such as the regulation of electron transfer, which helps to change the resistance to bacterial antibiotics (Silverman et al., 2003) and the rate of oxygen consumption (Kinkel et al., 2016). Therefore, bacterial NOS inhibition may provide a proper strategy to prevent bacterial colonization and infection. Although some noteworthy results were obtained, characterization and exploitation of the *NOS* gene, which may be used as an alternative antimicrobial target, will still be essential in future research. For example, it has been revealed that NOS has a pronounced effect on some biofilm-related genes; however, the types of mechanisms involved are confusing. In addition, the relationship between the *NOS* gene and bacterial oxidative stress should also be further explored. For example, more attention should be given to whether NOS has a potential effect on bacterial biofilm formation and drug resistance by oxidative stress.

This study has some limitations. SNPs are one of the most commonly used NO releasers in various related studies, and their physiological concentration and toxic concentration in bacteria have been reported in many studies (Williams et al., 2018). Therefore, we chose SNP as a typical representative of an NO releaser for the experiment. However, NO or the other NO donors DETA-NO, GEA 5024, and SNAP is used when biofilm formation is recommended for further confirmation of the effects of NO on biofilm biomass and planktonic biomass.

## Conclusion

In conclusion, our results demonstrate that the *NOS* gene in *S. epidermidis* can negatively regulate bacterial biofilm formation. Further study revealed that this *NOS* deletion was associated with bacterial growth, autolysis and biofilm dispersal. Therefore, our findings may provide a new approach for studying the pathogenesis of NOS in biofilm-associated infection of *S. epidermidis*.

## Data availability statement

The original contributions presented in the study are included in the article/**Supplementary Material**. Further inquiries can be directed to the corresponding author.

## Author contributions

JW was responsible for interpretation and drafting the manuscript. LR, ZH and LM participated in data acquisition and algorithms. TY, AS and ZY were responsible for data analysis. YG participated in its design and helped to draft the manuscript. All authors contributed to the article and approved the submitted version.

## Funding

This work was supported by Zhejiang Provincial Natural Science Foundation of China [grant number LY16H190006, LY18H160042]. Medical and Health Science and Technology Plan Project of Zhejiang Province [grant number 2021KY640, 2019KY278].

## Acknowledgments

We gratefully thank Prof. Di Qu and Dr. Yang Wu from Shanghai Medical College, Fudan University for providing the experimental design. We also thank Prof. Dazhi Jin from Hangzhou medical college for helpful comments in the preparation of this manuscript.

## Conflict of interest

The authors declare that the research was conducted in the absence of any commercial or financial relationships that could be construed as a potential conflict of interest.

The reviewer FY declared a past co-authorship with the author LR to the handling editor.

## Publisher’s note

All claims expressed in this article are solely those of the authors and do not necessarily represent those of their affiliated organizations, or those of the publisher, the editors and the reviewers. Any product that may be evaluated in this article, or claim that may be made by its manufacturer, is not guaranteed or endorsed by the publisher.

## Supplementary material

Supplemental Results Construction and Verification of NOS Gene Deletion and Complementation Strains. The NOS mutant of S. epidermidis was constructed through homologous recombination, as shown in **Figure 1A** of the supplementary materials. Without affecting the integrity of upstream and downstream sequences of the target gene, fragments of homologous arms of NOS were amplified, with sizes of 1032 and 1012 bp, respectively. The two fragments were ligated through fusion PCR, and the ligated fragments were then subjected to site-specific recombination with the pKOR1 plasmid to obtain the plasmid pKOR1-ΔNOS (**Figure 1A** of supplementary materials). Suspected deletion strains (SE1457-ΔNOS) were screened by the variable temperature. Primers were designed for PCR identification of the deletion strain and showed that the deletion strain was reduced by approximately 1758 bp compared with the wild-type strain (**Figure 1B** of the supplementary materials), indicating that the NOS gene was successfully deleted. The results were further verified through gene sequencing. Using 200 bp upstream of the NOS gene as a possible promoter region, website online prediction showed that the promoter region was 81-126 bp upstream of the NOS gene. The reverse primer design contained this region and the integral gene sequence. The NOS gene was amplified and ligated to the pRB473 plasmid after enzyme digestion with BamHI and EcoRI. The plasmid was electroporated into SE1457-ΔNOS, and a fragment of approximately 1223 bp was obtained after extraction, enzyme digestion and identification (**Figure 1C** of the supplementary materials), confirming that the complement strain was successfully constructed. The result was further confirmed through sequencing.

Supplementary Figure 1 Schematic map of gene deletion as well as construction and verification of deletion and complement strains. **(A)** Schematic diagram for the *NOS* gene deletion. **(B)** PCR verification for the *NOS* gene deletion. **(C)** PCR verification for the construction of the *NOS* gene complement strain. *SE1457*, *SE1457-ΔNOS* and *SE1457-ΔNOS:cNOS* are the wild strain, deletion strain and complement strain, respectively.
