# Supplementary figures and images for: The nitric oxide synthase gene negatively regulates biofilm formation in Staphylococcus epidermidis
Source: Front Cell Infect Microbiol. 2022 Nov 3;12:1015859. doi: 10.3389/fcimb.2022.1015859 (PMC9669438; doi:10.3389/fcimb.2022.1015859)

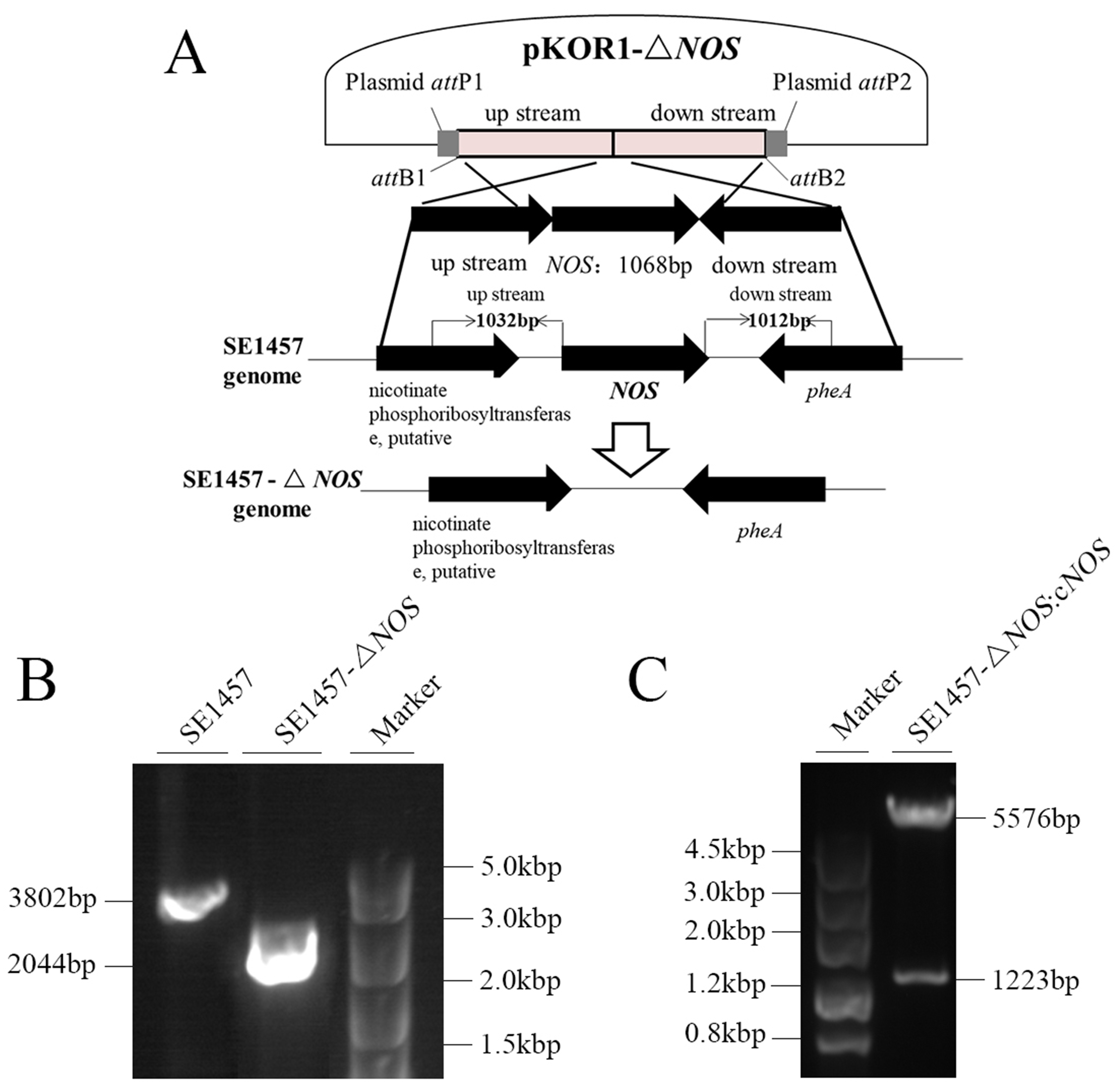

Supplement: Supplemental Results — Construction and Verification of NOS Gene Deletion and Complementation Strains. The NOS mutant of S. epidermidis was constructed through homologous recombination, as shown in Figure 1A of the supplementary materials. Without affecting the integrity of upstream and downstream sequences of the target gene, fragments of homologous arms of NOS were amplified, with sizes of 1032 and 1012 bp, respectively. The two fragments were ligated through fusion PCR, and the ligated fragments were then subjected to site-specific recombination with the pKOR1 plasmid to obtain the plasmid pKOR1-ΔNOS ( Figure 1A of supplementary materials). Suspected deletion strains (SE1457-ΔNOS) were screened by the variable temperature. Primers were designed for PCR identification of the deletion strain and showed that the deletion strain was reduced by approximately 1758 bp compared with the wild-type strain ( Figure 1B of the supplementary materials), indicating that the NOS gene was successfully deleted. The results were further verified through gene sequencing. Using 200 bp upstream of the NOS gene as a possible promoter region, website online prediction showed that the promoter region was 81-126 bp upstream of the NOS gene. The reverse primer design contained this region and the integral gene sequence. The NOS gene was amplified and ligated to the pRB473 plasmid after enzyme digestion with BamHI and EcoRI. The plasmid was electroporated into SE1457-ΔNOS, and a fragment of approximately 1223 bp was obtained after extraction, enzyme digestion and identification ( Figure 1C of the supplementary materials), confirming that the complement strain was successfully constructed. The result was further confirmed through sequencing. [file Image_1.jpeg]
